# ­Chemical profiling and biological activity of *Peperomia blanda* (Jacq.) Kunth

**DOI:** 10.7717/peerj.4839

**Published:** 2018-06-07

**Authors:** Wafa M. Al-Madhagi, Najihah Mohd Hashim, Nasser A. Awad Ali, Abeer A. Alhadi, Siti Nadiah Abdul Halim, Rozana Othman

**Affiliations:** 1 Department of Pharmacy, Faculty of Medicine, University of Malaya, Kuala Lumpur, Malaysia; 2 Department of Pharmacognosy, Faculty of pharmacy, Sana’a University, Sanaa, Yemen; 3 Drug Design and Development Research Group (DDDRG), University of Malaya, Kuala Lumpur, Malaysia; 4 Chemistry Department, Faculty of Science, University of Malaya, Kuala Lumpur, Malaysia; 5 Center of Natural Products Research and Drug Discovery (CENAR), University of Malaya, Kuala Lumpur, Malaysia; 6 Department of Pharmacognosy, Faculty of Clinical Pharmacy, Albaha University, Albaha, Kingdom of Saudia Arabia

**Keywords:** DPPH, FRAP, MTT, *Peperomia blanda*, TPC, Yemen

## Abstract

**Background:**

* Peperomia* belongs to the family of Piperaceae. It has different uses in folk medicine and contains rare compounds that have led to increased interest in this genus. *Peperomia blanda* (Jacq.) Kunth is used as an injury disinfectant by Yemeni people. In addition, the majority of Yemen’s population still depend on the traditional remedy for serious diseases such as cancer, inflammation and infection. Currently, there is a deficiency of scientific evidence with regards to the medicinal plants from Yemen. Therefore, this study was performed to assess the chemical profile and *in vitro* antioxidant and cytotoxic activities of *P. blanda.*

**Methods:**

Chemical profiling of *P. blanda* was carried out using gas chromatography mass spectrometry (GCMS) followed by isolation of bioactive compounds by column chromatography. DPPH• and FRAP assays were used to evaluate antioxidant activity and the MTT assay was performed to estimate the cytotoxicity activity against three cancer cell lines, namely MCF-7, HL-60 and WEHI-3, and three normal cell lines, MCF10A, WRL-68 and HDFa.

**Results:**

X-ray crystallographic data for peperomin A is reported for the first time here and *N,N*′-diphenethyloxamide was isolated for the first time from *Peperomia blanda*. Methanol and dichloromethane extracts showed high radical scavenging activity with an IC_50_ of 36.81 ± 0.09 µg/mL, followed by the dichloromethane extract at 61.78 ± 0.02 µg/mL, whereas the weak ferric reducing activity of *P. blanda* extracts ranging from 162.2 ± 0.80 to 381.5 ± 1.31 µg/mL were recorded. In addition, petroleum ether crude extract exhibited the highest cytotoxic activity against all the tested cancer cell lines with IC_50_ values of 9.54 ± 0.30, 4.30 ± 0.90 and 5.39 ± 0.34 µg/mL, respectively. Peperomin A and the isolated mixture of phytosterol (stigmasterol and β-sitosterol) exhibited cytotoxic activity against MCF-7 and WE-HI cell lines with an IC_50_ of (5.58 ± 0.47, 4.62 ± 0.03 µg/mL) and (8.94 ± 0.05, 9.84 ± 0.61 µg/mL), respectively, compared to a standard drug, taxol, that has IC_50_ values of 3.56 ± 0.34 and 1.90 ± 0.9 µg/mL, respectively.

**Conclusion:**

The activities of *P. blanda* extracts and isolated compounds recorded in this study underlines the potential that makes this plant a valuable source for further study on anticancer and antioxidant activities.

## Introduction

Years of research and discovery have proven that natural products have made significant contributions to new drug discovery for the treatment of cancer and infectious diseases, and valuable source of antioxidants (Moloney, 2016; Newman & Cragg, 2016).

There are numerous reactive species that are formed *in vivo* (classified as oxidising agents) that can lead to damaging DNA and biomolecules. Therefore, oxidative stress is considered as the main cause of development of degenerative diseases such as coronary heart disease, most cancers and ageing (Halliwell, 2007). In addition, various studies have confirmed that people deficient in antioxidative mechanisms are extra vulnerable to intense bacterial and fungal infections and HIV (Nathan & Shiloh, 2000; Rahal et al., 2014).

Cancer was classed as one cause of morbidity and mortality in 2012, affecting populations in all countries (McGuire, 2016). Breast cancer is considered the most prevalent among women, whereas leukaemia is the most prevalent in those 12–25 years old. The main complication of leukaemia is infection (Holliday & Speirs, 2011; Levenson & Jordan, 1997). Therefore, cancer chemotherapy has become a major focus of research, worldwide. Natural products are important source for drug development due to the complex molecular structures of the active compounds, and they have the ability to interact with mammalian cell targets.

Piperaceae has a widely distributed pantropical family. The largest genera in the family are *Piper* with 2,000 species and *Peperomia* with 1,700 species (Gutierrez et al., 2016). *Peperomia* has various uses in folk medicines, such as for the treatment of inflammation, gastric ulcers, asthma, pain and bacterial infection (Coseri, 2009; Felippe et al., 2008; Oloyede, Onocha & Olaniran, 2011; Salamah & Hanifah, 2014; Xu et al., 2006). Moreover, previous studies have reported *Peperomia* species to be rich in secondary metabolites. For example, twenty seven compounds were isolated from *P. vulcanica* and *P. fernandopoioana (Mbah et al., 2012),* nineteen compounds from *P. sui* (Cheng & Chen, 2008) and three novel secolignans from *P. blanda* (cultivated in Brazil) (Felippe et al., 2011). In addition, sterols, two chromones and two C-glycosyl flavones were previously isolated from *P. blanda* (Velozo et al., 2009) and showed modest antioxidant activity. Furthermore, two chromenes, separated from the aerial parts of the species, and tetrahydrofuran lignan have also been shown to have anti-trypanosomal properties. Therefore, presence of a large number of rare secolignans (peperomins, chromenes and polyketides of 2-acyl-cyclohexane-1,3-dione) make this genus a source of unique compounds.

*Peperomia blanda* is a herb species that is naturally distributed in Madagascar, Yemen, Macarena Island, Taiwan, Polynesia, Australia and also in Florida, USA to South America (Mathieu et al., 2011). *P. blanda,* cultivated on Socotra *Island* (Yemen), remains poorly studied. In this regard, the present study was designed to determine the chemical composition of *Peperomia blanda* (Jacq.) Kunth through the isolation of active components and assessment of their *in vitro* cytotoxic and antioxidant activities.

## Material and Method

### Plant collection and extraction

*Peperomia blanda* (Jacq.) Kunth was collected from the Socotra Island, Yemen (Diksam-sikand), with approval number 120025 and, 120026 from the General Directorate of Plant Protection, Yemen, and identified by Dr. Abdul Wali Al-Khulaidi, Department of Botany, Taiz University. The voucher specimen number PPLS/2013/90/1 was placed at the Faculty of Pharmacy, Sana’a University. Plant samples were oven-dried for 7 days at 30 °C. The dried sample was milled and stored at room temperature for further analysis. Dried plant sample weighing 50 g was successively extracted by Soxhlet extraction using solvents with different polarities, starting with non-polar petroleum ether, then dichloromethane and methanol. The resulting solvent extracts were concentrated under reduced pressure to obtain crude extracts for further biological studies and compound isolation. All solvents used were of analytical grade (Merck, Darmstadt, Germany).

### Chemical profiling determination

Chemical profiling of the different solvent extracts was carried out using gas chromatography mass spectrometer (Narayanamoorthi, Vasantha & Maruthasalam, 2015) QP2010 Plus (Shimadzu, Kyoto, Japan) with an auto sampler. The petroleum ether and dichloromethane extracts were dissolved in chloroform, and the methanol extract was dissolved in methanol, and 1 µL of each sample (100 µg/mL) was added to the column. A fused HP5-MS silica capillary column (30 m × 0.32 mm, film thickness 0.25 µm) was used. The carrier gas was helium, and 1:50 split ratio was used. The oven temperature was maintained at 50 °C for 5 min, after which it was slowly increased at a rate of 5 °C per min to 240 °C and maintained at 240 °C for 5 min. The injection port temperature was 250 °C and the spectra of the constituents of the sample solution were matched with the spectra of identified compounds kept in the internal library (Wiley; mass spectral library).

### Isolation of compounds

The *in vitro* cytotoxic and antioxidant assays of the crude solvent extracts were evaluated and showed activities. Therefore, to isolate bioactive compounds, petroleum ether and dichloromethane extracts were fractionated using column chromatography (50 × 3.5 cm) on silica gel and run with solvent gradients of chloroform:EtOAc (100:0, 95:5, 90:10, 85:5, 80:20, 75:25, 70:30 until 0:100 %v/v, each 400 mL), EtOAc:MeOH (100:0, 95:5, 90:10, 85:5, 80:20, 75:25, 70:30 until 0:100 %v/v, each 400 mL), and finally with 800 mL MeOH. A total of 200 sub-fractions (50 mL) were collected based on their TLC (8:2 of CHCl3:EtOAc, solvent system) into four main fractions of petroleum ether and three fractions of dichloromethane. Fraction 2 of the petroleum ether extract was further chromatographed with an n-hexane:EtOAc solvent system on silica gel to afford small needle crystals of *N,N*′-diphenethyloxamide and phytosterol mixture (stigmasterol and β-sitosterol). Dichloromethane fraction 2 was subjected to silica gel column chromatography for fractionation and purification using the gradient elution method with an n-hexane:EtOAc solvent system. Recrystallization was done by dissolving crystal in small amount of ethyl acetate and drops of hexane were added till turbid solution start to appear. Then solution left to dry and crystal start to form.

### Structural elucidation

Chemical structures of isolated compounds were elucidated using FT-IR/FT-FIR (Perkalin), GCMS (QP2010 Plus, Shimadzu, Kyoto, Japan) and 1D and 2D NMR (Nuclear Magnetic Resonance, JEOL, Peabody, MA, USA) spectroscopy at 500 MHz with deuterated chloroform as solvent. The crystal compounds were identified via single crystal X-ray structure determination (Oxford Diffraction Xcalibur Gemini S diffractometer; Agilent, Santa Clara, CA, USA) equipped with CuKα radiation, (*k* = 1.5418 Å). The data were processed using CrysAlis software and Empirical absorption correction using spherical harmonics was implemented using SCALE3 ABSPACK scaling algorithm (Xcalibur, 2007). The figures were produced using MERCURY software (Macrae et al., 2008).

### Physical, chemical and spectroscopic data of the compounds

#### Peperomin A

White crystal, M.P = 154–157 °C. UV ^CHCl3^*λ*_max_ nm: 333, 294, 311. IR *ν*_max_ (CHCl_3_): 2958, 2870 (C-H), 1667 (C=C), 1462, 1389, 1220, 1190 (C-H) cm^−1^. The X-ray diffraction analysis, molecular formula (C_22_H_22_O_8_); EI-MS m/z: 414 (35), 315 (100), 299 (4), 285 (5). ^1^HNMR (CDCl_3_, 500 MHz) *δ* ppm: 6.41 (1H, *d, J* = 1*.95 Hz*, H-6′,6″), 6.33 (1H, *d, J* = 1*.95* Hz, H-2′,2″), 5.90-5.92 (2H, *d*, *J* = 2.00 Hz, 2 × OCH_2_O), 4.27 (1H, *m*, H-4a), 3.87 (3H, *s*, 2 × OCH_3_), 3.76 (1H, *m*, H-4b), 3.54 (1H, *d*, *J* = 10.9 Hz, H-5), 2.83 (1H, *m*, H-3), 2.31 (1H, *m*, H-2), 0.90 (3H, *d, J* = 8*.05* Hz, H-6). ^13^CNMR (CDCI_3_, 125MHz) *δ* ppm: 179.6 (C-1), 149.4 (C-3′), 149.3 (C-3″), 143.5 (C-5’), 143.6 (C-5″), 134.3 (C-4′), 136.1 (C-4″), 134. 4 (C-1′), 136.7 (C-1″), 107.7 (C-6′), 107.7 (C-6″), 101.6 (C-2′), 101.6 (C-2″), 101.1 (OCH_2_O), 101.3 (OCH_2_O), 70.3 (C-4), 56.0 (2 × OCH_3_), 56.2 (C-5), 47.1 (C-2), 40.2 (C-3), 15.8 (C-6).

#### *N, N*′-diphenethyloxamide

Crystal needle. M.P = 184–185 °C UV ^CHCl3^*λ*_max_ nm: 290, 382. IR *ν*_max_ (CHCl_3_): 2993, 1635 (C=O), 1459, 1044 (C-H) 1364 (C-N). UPLC/Q-TOF-MS m/z [M+1]^+^ 296.300 (13.08), 235.1300 (25), 209.1200 (19), 123.0700 (14) (calcd for C_18_H_20_N_2_O_2_, 296.3628). ^1^HNMR (CDCl_3_, 500 MHz) *δ* ppm: 2.24 (2H, *m*, H-), 3.50 (2H, *m*, H-1′), 3.50 (1H, *m*, H-2′), 7.22 (3H, *m*, H-Ar). The X-ray diffraction analysis gave molecular formula (C_18_H_20_N_2_O_2_) and molecular mass 296.3 g/mol.

#### Stigmasterol and β-sitosterol

White powder, percent of yield (0.04%), IR *ν*_max_ (CHCl_3_): 3200 (O–H,), 2870(C-H), 1667 (C=C), 1462, 1389, 1363, 1311, 1220, 1190 (C-H) cm-1. GCMS m/z 414.12 and 412.52 [M+1]^+^ (calc. for C_29_H_48_O = 412.69) (100), 351 (30), 255 (50), 55 (85) and (C29H50O=414.71) (40), 57(70), 43(100). ^1^HNMR (CDCl_3_, 500 MHz) *δ* ppm: 0.63 (3H, *s*, H-28), 5.14 (2H, *t*, *J* = 5.15 Hz, H-21), 4.98 (2H, *m*, H-20), 3.46 (1H, *m*, H-3), 1.19 (3H, *s*, H-29), 0.99 (3H, *d*, *J* = 2.85 Hz, H-19), 0.85 (3H, *t*, *J* = 5.15 Hz, H-24), 0.76 (3H, *d*, *J* = 2.9 Hz, H-26), 0.75 (3H, *d*, *J* = 1.15 Hz, H-27). ^13^C NMR (CDCl_3_, 125 MHz,) *δ* ppm: 140.8 (C-5), 138.4 (C-20), 129.3 (C-21), 121.8 (C-6), 71.8 (C-3), 56.8 (C-14), 56.0 (C-17), 50.2 (C-9), 45.8 (C-22), 42.2 (C-4), 40.6 (C-18), 39.7 (C-12), 36.5 (C-1), 31.9 (C-2), 31.7 (C-8), 30.3 (C-10), 30.3 (C-7), 29.2 (C-25), 29.0 (C-16), 25.5 (C-23), 24.4 (C-15), 21.1 (C-26), 21.1 (C-19), 19.4 (C-27), 18.8 (C-28), 12.1 (C-29), 12.0 (C-24).

### Biological activity

#### Cytotoxicity assay using the colorimetric assay

*In vitro* cytotoxic potency of the investigated extracts was established using the colorimetric (MTT) assay. The three cancer cell lines involved were the human breast adenocarcinoma (MCF-7), murine myelomonocytic leukaemia (WEHI-3) and human promyelocytic leukaemia (HL-60). The three normal cell lines used were the human mammary epithelial cell line (MCF10A), primary dermal fibroblasts cell line (HDFa) and the human hepatic cell line (WRL-68). All cells were from American Type Culture Collection (ATCC) and were subcultured in tissue culture flask (75 cm) with RPMI1640 media mixed with 10% fetal bovine serum and 1% penicillin–streptomycin and kept in an incubator at 5% of CO_2_ saturation and 37 °C. The following solutions were prepared: crude extracts (dissolved in 1% DMSO) at various concentrations (100, 50, 25, 12.5, 6.3, 3.5, 1.5 and 0 µg/mL); and peperomin A, phytosterol mixture and standard drugs (Taxol and vinblastine) as positive controls at decreasing concentrations (50, 25, 12.5, 6.3, 3.5, 1.5, and 0 µg/mL). *N,N*′-diphenethyloxamide was not involved in the biological assay because of the low yield obtained. Extracts were incubated with the cancer cell lines for 72 h, and peperomin A and the phytosterol mixture were incubated for 24, 48 and 72 h. After the incubation periods, 20 µL of MTT solution (5 mg/mL) was added to each well and incubated for 4 h. To dissolve the formazan crystals formed, 100 µL of DMSO was added to each well. The IC_50_ values were calculated from the plot of cancer cell growth versus the concentration of extracts/compounds.

#### Antioxidant assays

##### Free radical scavenging activity.

The free radical scavenging activities of the extracts, peperomin A and phytosterol mixture were estimated using the 1,1-diphenyl-2-picryl-hydrazyl (DPPH) assay. Quantitative estimation was done in 96-well ELISA plates in triplicate. Complete of 70 µL of DMSO was brought for each well followed by 100 µL of plant extract solution or isolated tested compounds. Various concentrations of each extract and peperomin A (15.6 –1,000 µg/mL) were used. Similar concentrations of ascorbic acid were used as positive control. A total of 30 µL DPPH (3.14 X 10^−3^ µM) was added to each well, then the plate was allowed to stand in the dark at 25 °C for 30 min, and the absorbance was later recorded taken at 517 nm. The free radical scavenging activity was calculated using the following equation:

\begin{eqnarray*}\text{Free radical scavenging activity}(\text{%})=\nonumber\\\displaystyle \quad [(\text{Abs of DPPH}-\text{Abs of sample})/\text{Abs of DPPH}]\times 100\text{%}. \end{eqnarray*}

where Abs is absorbance. The relationship between each concentration and its scavenging percentage was plotted, and the IC_50_ value was calculated by interpolation. Scavenging activity was represented by IC_50_ values (the extract concentration that scavenges 50% of DPPH radicals).

#### FRAP assay

The antioxidant power of *P. blanda* extracts by ferric reduction was done by preparing fresh FRAP reagent from acetate buffer (pH 3.6), a solution of 10 mM TPTZ (2,4,6-tri(2-pyridyl)-s-triazine) in 40 mM HCl and a solution of 20 mM iron (III) chloride in a ratio of 10:1:1 (%v/v) (Brand-Williams, Cuvelier & Berset, 1995; Mohammadzadeh et al., 2007; Mothana et al., 2009; Salazar-Aranda et al., 2011), butylated hydroxytoluene (BHT), and ascorbic acid were used as controls. Then, 10 µL of plant extracts, peperomin A, ferrous sulfate (standard) and the controls were added to 300 µL FRAP reagent (triplicate) in the dark and the absorbance was measured at 593 nm. A linear standard curve was constructed using 100 and 1,000 mM FeSO_4_. The results were represented as the ratio of the concentration of iron(III) mM over the extract dry weight (g).

### Total phenolic and flavonoid contents determination

The total phenolic contents extracted were estimated by the Folin–Ciocalteu reagent (Narayanamoorthi, Vasantha & Maruthasalam, 2015; Wei et al., 2011). A seven-point standard curve (0–50 mg/L) for gallic acid (≥ 97%, GA) was built to calculate total phenolic percent as Gallic acid equivalent (mg GA/g dry extract). Analyses were performed in triplicates. The extract total flavonoid contents were evaluated based on the aluminum chloride colorimetric method (Wei et al., 2011; Xcalibur, 2007), using quercetin as standard. Seven-point standard curve (0–1,000 mg/L) of quercetin was used and the levels of extracts total flavonoid content **were** calculated as quercetin equivalent (mg quercetin/g dry extract).

## Results and Discussion

Screening of plants for their medicinal values remains an important area in scientific research, and from the 1940s to 2014, 75% of the clinically approved small molecules originated, or were directly derived, from natural products (Newman & Cragg, 2016). Therefore, in this study, *in vitro* cytotoxic and antioxidant activities of *P. blanda* were evaluated. Chemical profiling of crude solvent extracts was performed using GCMS, followed by isolation of bioactive compounds via column chromatography.

**Figure 1:**
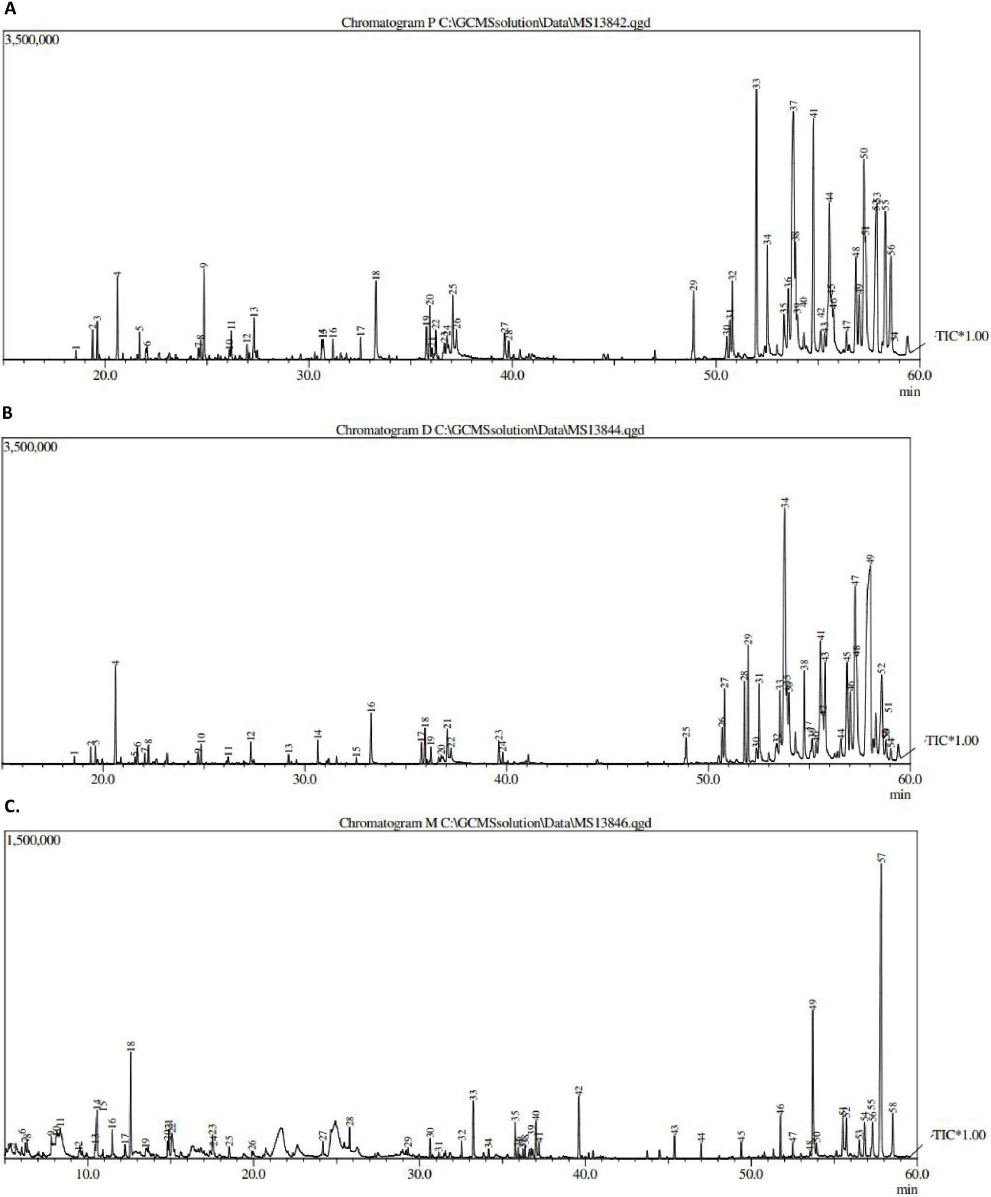
GCMS chromatogram of (A) pet. ether extract, (B) DCM extract and (C) MeOH extract.

### Chemical profiling

The results obtained from GCMS investigation of the crude extracts led to the identification of several compounds. The different extracts showed 53–57 peaks (Fig. 1).

The major peaks are summarized in Table 1 and the most commonly identified compounds were fatty acids, sesquiterpenes, palmitic acid, vitamins and steroids. Those compounds have been reported to have diverse biological activities, including antioxidant, antimicrobial, anticancer and anti-inflammatory activities (Narayanamoorthi, Vasantha & Maruthasalam, 2015). In addition, hydroquinone was identified, and has been reported to be used for the treatment of hyperpigmentation.

**Table 1:** The major compounds detected in the different extracts of *P. blanda* by GCMS analysis.

Extracts	RT Minute	Name of compound	Molecular formula	Molecular weight (g/mol)	Peak area %	Compound nature	Activity
MeOH	12.584	4H-pyran-4-one	C_6_H_8_O_4_	144	5.05	Central core of flavanoid	
	33.238	n-Hexadecanoic acid	C_16_H_32_O_2_	256	2.39	Palmitic acid	Antioxidant, Hypocholesterolemic, Nematicide, lubricant, flavor
	39.602	n-Nonadecanol-1	C_19_H_40_O	284	2.83	Fatty alcohol	Antibacterial, anti-tubercular and cytotoxic activities
	53.701	iso-propyl 5,9-hexacosadienoate	C_29_H_54_O_2_	392	8.07	Fatty acid	Antimicrobial
	57.825	Pyrazole,(3-furyl)-1-(4-nitrophenyl)-5-phenyl-	C_6_H_8_O_4_	331	24.92	Heterocyclic compound	
	20.618	Caryophyllene	C_15_H_24_	204	2.18	Sesquiterpene	Anti-tumor, Analgesic, Anti-bacterial, Anti-inflammatory Sedative, Fungicide
	33.281	n-Hexadecanoic acid	C_15_H_24_O	256	1.25	Palmitic acid	Antioxidant, Hypocholesterolemic, Nematicide, lubricant, flavor
	50.799	2-cyclohexen-3,6diol-1-one2-tetradeconoyl	C_17_H_36_O	338	1.86		
	51.964	Tetrapentacontane	C_17_H_32_O_2_	758	2.39	Higher alkane	Lubricant, beewax
DCM	53.787	(Phenylthio)acetic acid, hexadecyl ester	C_54_H_11_O	392	14.25		
	55.556	Phen-1,3-diol,2-dodecanoyl	C_24_H_40_O_2_S	292	4.67	Hydroquinone	Treatment of hyperpigmentation
	57.273	(Phenylthio)acetic acid, octadecyl ester	C_60_H_122_	420	7.42		
	58.024	Vitamin E	C_18_H_28_O_3_	430	20.8	Vitamin compound	Antinflammatory, Antioxidant, Antidermatitic, Antileukemic, Antitumor, Anticancer
	58.585	Gamma-sitosterol	C_29_H_50_O	414	4.61	Steroid	Hepatoprotective Antiasthma, Anti inflammatory Diuretic, Cancer preventive Antioxidant
	20.605	Cyclohexane, 1-ethenyl-1-methyl-2,4-bis(1-methylethenyl)-	C_15_H_24_	204	1.81	Sesquiterpene	Anti-tumor, Analgesic, Anti-bacterial, Anti-inflammatory, Sedative, Fungicide
	24.849	caryophyllene oxide	C_15_H_24_O	220	1.73	Sesquiterpene	
	33.293	1-Hexadecanol, 2-methyl-	C_17_H_36_O	256	1.31	Cetyl alcohol	Antimicrobial
	36.233	Phytol	C_20_H_40_O	296	2.82	Diterpene	A manufacture precursor of synthetic form of vitamin E
	37.073	cis-10-Heptadecenoic acid	C_17_H_32_O_2_	268	1.31	Acetate	No activity reported
	51.979	Teterapentcontane	C_54_H_11_O	758	6.54	Sesquiterpene	Anti-inflammatory and Antioxidant activities
Pet. ether	53.789	(Phenylthio)acetic acid, hexadecyl ester	C_29_H_50_O	392	12.81		
	54.773	Hexacontane	C_15_H_24_	842	7.29	Saturated hydrocarbon	
	55.561	Phen-1,3-diol,2-dodecanoyl	C_15_H_24_O	292	5.71	Hydroquinone	Reducing agent, antioxidant
	57.253	Tetrapentacontane-1,40-diol	C_17_H_36_O	420	7.5	Saponin	Fragrance compounds
	57.33	*β*-Sitosterol	C_29_H_48_O	412	4.24	steroid	
	57.895	Vitamin E	C_17_H_32_O_2_	430	2.81	Vitamin	Antidiabetic, Anti-inflammatory, Antioxidant, Antileukemic, Antitumor, Anticancer,
	58.308	Teterapentcontane	C_54_H_11_O	758	4.87	Sesquiterpene	Anti-inflammatory and antioxidant activities
	58.58	Stigmasterol	C_29_H_50_O	414	6.10		

It can be seen from the data of GCMS in Table 1 that the presence of *N,N*′-diphenethyloxamide, *β*-sitosterol and stigmasterol with molecular ion peaks at 296, 414 and 412 (m/z), respectively, could be detected in petroleum ether with (peak number, retention time (RT)) values of (22, 36.23), (52, 57.33) and (56, 58.58) min. Moreover, GCMS analysis of the dichloromethane extract showed the presence of peperomin A with a molecular ion peak, peak number and RT of 414, 52 and 58.58 min, respectively.

### Isolation of compounds

**Figure 2:**
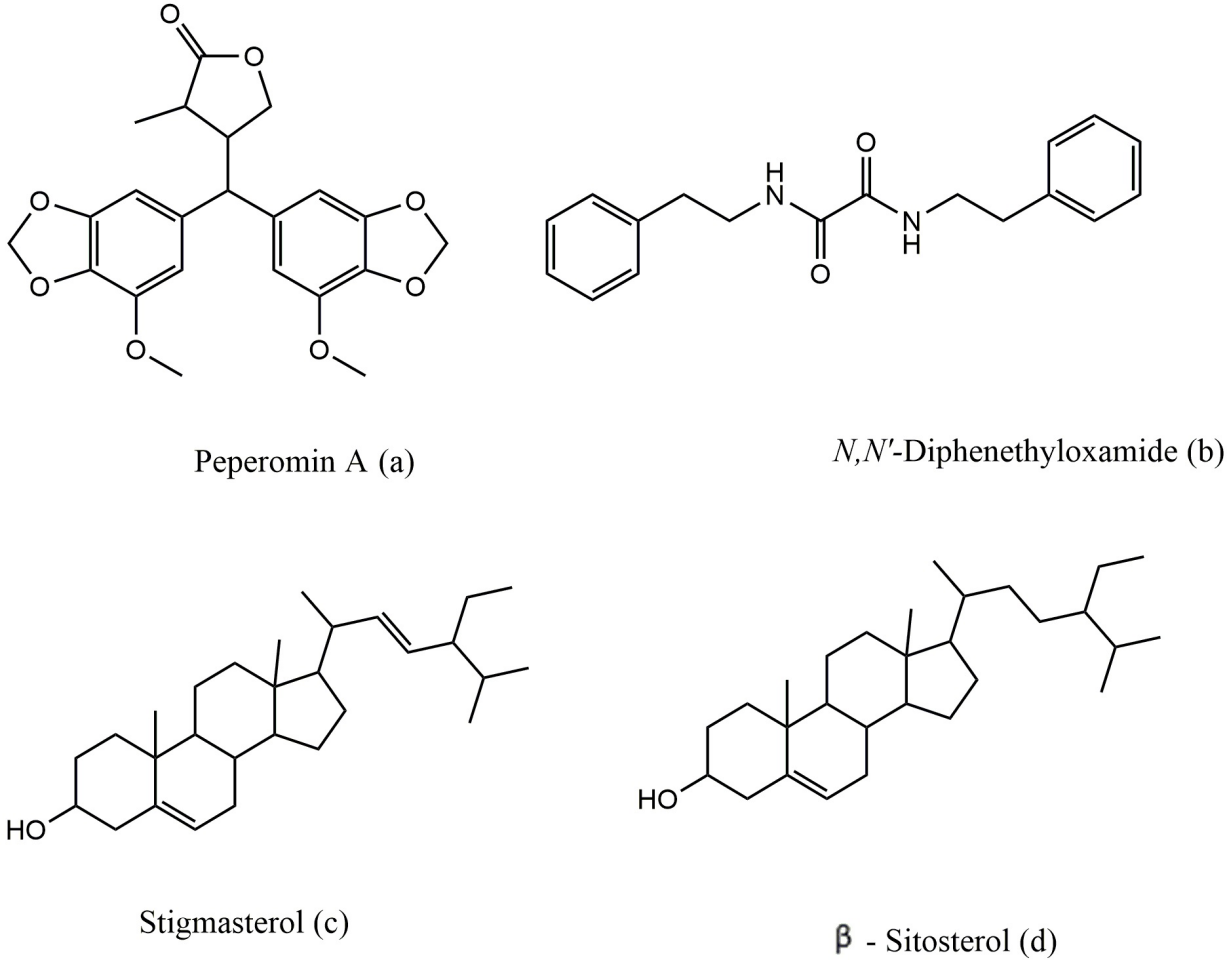
The chemical structure of peperomin A (A), N,N′-diphenethyloxamide (B), Stigmasterol (C) and β-sitosterol (D).

The active petroleum ether and dichloromethane extracts of *P. blanda* were repeatedly chromatographed on silica gel and this successfully led to the isolation of three compounds (Fig. 2), and the chemical structures were elucidated using 1D and 2D NMR, GCMS and X-ray diffraction for crystals obtained.

### Single crystal X-ray structure determination

Peperomin A was isolated in crystal form for the first time, and the X-ray crystal structure was deposited with the Cambridge Crystallographic Data Centre (CCDC) with deposition number CCDC 1529063. The structure of peperomin A was resolved in the centrosymmetric triclinic space group P 2_1_ 2_1_ 2_1_, with one molecule in the asymmetric unit (Table 2). Lattice parameters have been determined as 11.3200 Å, b 11.6312 Å, c 14.5155 Å and the volume of the unit cell is found to be 1911.19 Å^3^. R-configuration is present at the chiral center C3 and S-configuration at the chiral center C5 of 2-methyl-butyl lactone ring. The x-ray molecular structure of peperomin A showed the same information found in solution by NMR spectroscopy that were also comparable with data reported by Felippe et al. (2011).

**Table 2:** Crystal data and structure refinement for peperomin A and N,N′-Diphenethyloxamide.

	Peperomin A	*N,N*′-Diphenethyloxamide
Molecular formula	C_22_H_22_O_8_	C_18_H_20_N_2_O_2_
Formula weight g/mol	414.40	296.36
Temperature	293 K	293 K
Wavelength	1.54178Å	1.54178 Å
Crystal system	Orthorhombic	Monoclinic
Space group	P2_1_2_1_2	P2_1_/c
Unit cell parameters:		
*a/* Å	11.3200(2)	10.6818(14)
*b/* Å	11.6312(2)	5.1688 (6)
c/ Å	14.5155 (3)	14.248 (2)
*α*/°	90	90
*β*/°	90	97.5(13)
*γ*/°	90	90
Volume (Å^3^)	1911.19(6)	779.90(18)
Z	4	2
Density (calculated)/gcm^−3^	1.440	1.262
Absorption coefficient./µmm^−1^	0.926	0.663
Crystal size (mm)	0.3 × 0.25 × 0.25	0.3 × 0.25 × 0.25
Θ limits/°	4.0370–36.3	4.5–37.5
F (0000)	872	316
Index ranges	−13 ≤ h ≤ 14, − 14 ≤ k ≤ 13, −12 ≤ 1 ≤ 17	−13 ≤ h ≤ 12, −6 ≤ k ≤ 6, −16 ≤ 1 ≤ 17
Reflection collected	5424	2793
Independent reflections	3340 [R_int_ = 0.0163]	1554 [R_int_ = 0.0436]
Completeness to Θ = 36.3°	98.0 %	96.6 %
Data /restrains /parameters	3340∕0∕271	1554∕0∕100
Goodness of fit on F2	1.062	1.038
R for I >2 *α* (I)	R1 = 0.0318	R1 = 0.1091
R for all data	wR2 = 0.0803	wR2 = 0.2219
Δ*ρ*_min_/e A^−3^	−0.231	−0.483
Δ*ρ*_max_/e A^−3^	0.234	0.336

From 1D and 2D NMR data, it is apparent that ^1^H NMR spectrum displayed resonances of four meta-coupled aromatic hydrogens at 6.41 (H-6′,6″, *d*, *J* = 1.95 Hz), 6.33 (H-2′,2″, *J* = 1.5 Hz), two methylenedioxy groups at 5.90–5.92 (*d*, *J* = 1.2 Hz), and two O-methyl singlets at 3.87. This set of signals characterized two 3,4-methylenedioxy-5-methoxy phenyl rings with magnetically non-equivalent methylenedioxy protons as a result of the anisotrophy from aromatic ring (Felippe et al., 2011). A doublet at *δ* 0.90 (H-6, *d*, *J* = 8.0 Hz) was assigned to the methyl group, and multiplets at 3.76 (H-4a) and 4.27 (H-4b) were due to the methylene group of the butyrolactone moiety. The resonances at *δ* 2.31 (H-2, m), 2.83 (H-3, m), and 3.54 (H-5, *d*, *J* = 10.9 Hz), due to three methine groups, were then observed. A Ϩ-butyrolactone ring was confirmed via HMBC between H-2, H- 4, and H-6 and the lactone carbonyl carbon at 179.9 ppm (C-1). The ^13^CNMR data corroborated the presence of the butyrolactone system, and all signals involved were accordingly assigned based on the HMBC data.

**Figure 3:**
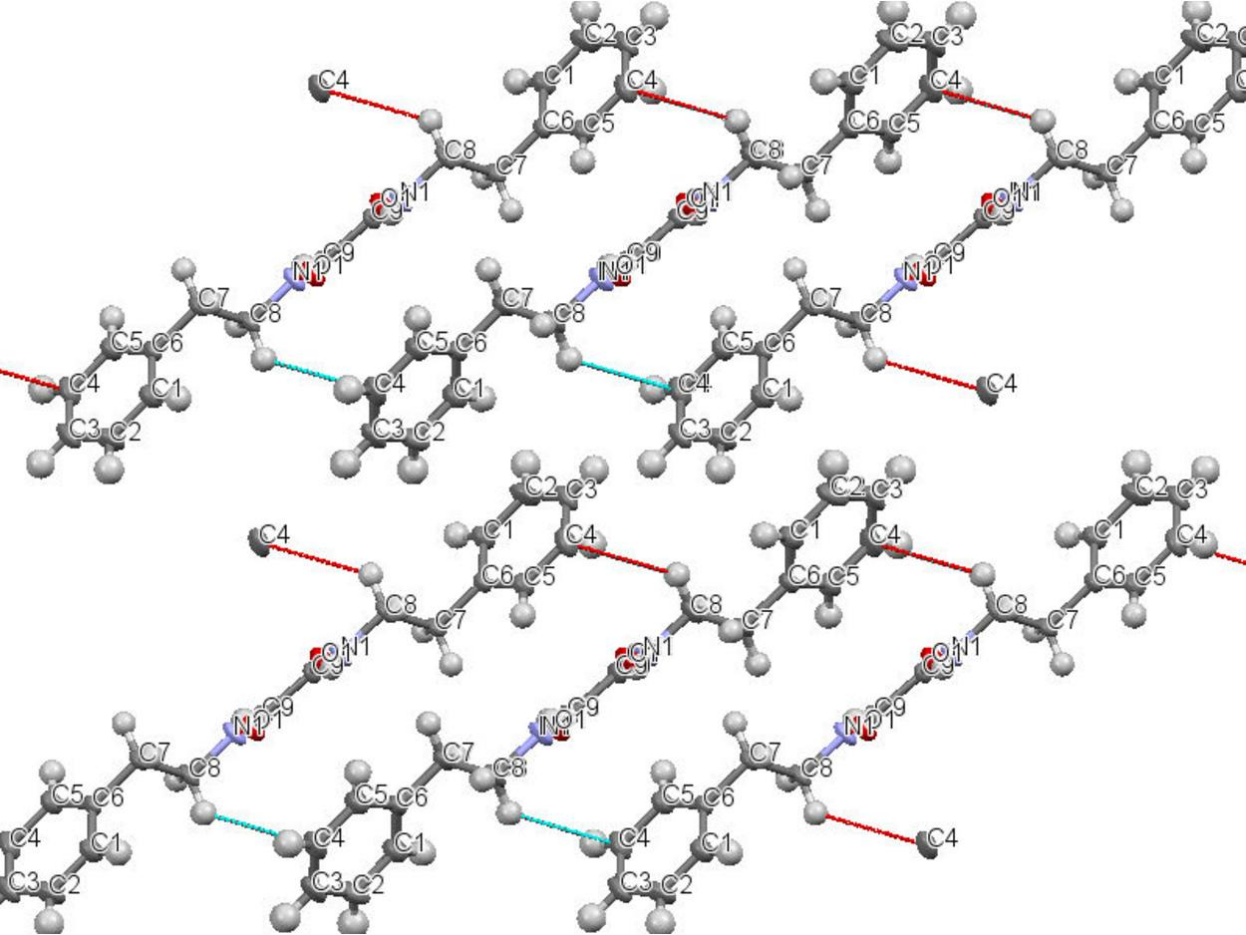
Intermolecular interaction in the crystal structure packing of *N,N*′-diphenethyloxamide.

*N,N*′-diphenethyloxamide is a symmetric oxamide that have synthesized before to serve as a simple model for hydrogen bonding interaction that could be recoded in peptides and proteins (Martínez-Martínez et al., 1998). However to our knowledge, this is first time that it is isolated from the natural source. In this study, *N,N*′-diphenethyloxamide is the second crystal isolated for the first time from *Peperomia* species and the crystal structure was deposited with the CCDC with deposition number CCDC 1529062. The structure of *N,N*′-diphenethyloxamide (Fig. 2) was resolved in the centrosymmetric monoclinic space group P 2_1_/c, as summarized in Table 2. The crystal oxamide group has two independent amides that disregard a *π*-conjugation through the C-C central bond, as shown by the average bond length of the oxalyl (OC-CO) of 1.2306 Å and torsion angle value of −180.0 °, in comparison to the oxalyl (OC-CO) average bond length of 1.541 Å and torsion angle of 180.0 for a trans conformation of *N,N*′-bis(2-hydroxyphenyl)oxamide and torsional angle between 90–115° for other oxamide derivatives reported previously (Martínez-Martínez et al., 1998). In crystal packing, the nitrogen atoms (N and N′) of oxamide was hydrogen bonded to the oxygen atom of the carboxylic acids through N-H⋯O intermolecular interaction establishing a sheet along the axes (Fig. 3) and the diffraction data details are summarized in Table 2.

The data for ^1^HNMR chemical shifts of Ar-CH_2_ and, CH_2_-N appeared at 2.24 and 3.50 ppm, and aryl hydrogen signals that are found at 7.22 ppm that are supported by previous data recorded for the synthesis of *N,N*′-diphenethyloxamide (Martínez-Martínez et al., 1998). UV absorption bands were obtained at 290, and 382 nm. The IR spectrum recorded the presence of different bands at 2993 to indicate the presence of NH group, and 1635 (C=O), 1459, 1044 (C-H) 1364 (C-N). These results are consistent with the data obtained from IR spectrum of synthesized *N,N*′-diphenethyloxamide that recorded IR *ν*_max_ (KBr)/cm^−1^ at 3,305, 1,653, 1,520, 1,495, 1,224, 1,189 and 697. Another important finding was the peak record from; UPLC/Q-TOF-MS m/z [M+1]^+^ at 296.300 for C_18_H_20_N_2_O_2_. The X-ray molecular structure of *N,N*′-diphenethyloxamide supported that data obtained from ^1^HNMR, IR, mass spectroscopy and the previous recorded study for the synthesized *N,N*′-diphenethyloxamide.

### Biological activity

Preliminary screening of the extracts using MTT assay showed cytotoxic activity against different cell lines. The petroleum ether extract exhibited IC_50_ values of 9.54 ± 0.30 and 4.3 ± 0.90 µg/mL against HL-60 and WEHI-3 cells, respectively. The dichloromethane extract showed IC_50_ values of 14.42 ± 1.52 and 15.58 ± 1.17 µg/mL against HL-60 and WEHI-3 cells, respectively. The methanol extract showed lower activity against HL-60 and WEHI-3 cell lines. However, methanol and petroleum ether extracts showed promising activity against MCF-7, with IC_50_ values of 10.49  ± 0.79 and 5.39 ± 0.34 µg/mL, respectively. The extracts showed low cytotoxicity against normal cells (MCF10A, WRL-68 and HDFa), with IC_50_ values greater than 100 µg/mL (Table 3).

**Table 3:** IC_50_ (µ/mL) values of different extracts against various human cell lines.

Cell line	Pet. Ether extract	DCM extract	MeOH extract	Vinblastine	Paclitaxel
	72 hr				
WEHI-3	4.3 ± 0.90	15.58 ± 1.17	104.39 ± 1.25	0.57 ± 0.035	ND
HL-60	9.54 ± 0.30	14.42 ± 1.52	28.62 ± 2.19	5.00 ± 0.01	ND
MCF-7	5.39 ± 0.34 (18.5)^a^	30.56 ± 0.84 (3.2)^a^	10.49 ± 0.79 (9.5)^a^	ND	3.00 ± 0.07 (33.3)^a^
WRL68	>100	>100	>100	ND	ND
HDFa	>100	>100	>100	ND	ND
MCF10A	>100	>100	>100	ND	ND

**Notes.**

a Ratio of the mean IC_50_ value deterfmined in the normal MCF10a cell line over the mean IC_50_ value determined in MCF-7 cancer cell line.

Only the selectivity indexes greater than one are referenced.

ND not determined

**Table 4:** IC_50_ values of Peperomin A and phytosterol against various human cell lines.

Cell lines	Peperomin A			Phytosterol			Vinblastine			Paclitaxel		
	24 hr	48hr	72hr	24 hr	48hr	72hr	24 hr	48hr	72hr	24 hr	48hr	72hr
WEHI-3	20.09 ± 0.46	18.68 ± 0.73	4.62 ± 0.03	18.04 ± 0.58	12.93 ± 0.80	8.06 ± 0.16	4.11 ± 0.06	3.56 ± 0.07	0.57 ± 0.04	3.16 ± 0.02	2.15 ± 0.02	1.90 ± 0.9
HL-60	43.62 ± 0.10	26.34 ± 0.26	16.36 ± 0.62	29.47 ± 0.80	13.29 ± 0.37	9.84 ± 0.61	5.91 ± 0.31	4.81 ± 0.19	2.36 ± 0.08	12.73 ± 0.40	8.67 ± 0.76	5.13 ± 0.32
MCF-7	40.73 ± 0.92 (1.1)	9.22 ± 0.28 (1.3)	5.58 ± 0.47 (7.4)	30.69 ± 0.81 (1.62) b	15.21 ± 0.81 (3.28) b	8.94 ± 0.05 (5.59) b	14.58 ± 0.16	7.57 ± 0.50	5.16 ± 0.42	5.87 ± 0.19	4.47 ± 0.03	3.56 ± 0.34
WRL68	>50	>50	41.5 ± 0.54	>50	40.94 ± 0.29	37.27 ± 1.5	>50	>50	>50	>50	>50	>50
HDFa	>50	>50	>50	>50	>50	49.42 ± 0.56	>50	>50	>50	>50	>50	>50
MCF10A	>50	>50	>50	>50	>50	>50	>50	>50	>50	>50	>50	>50

**Notes.**

Mean and (standard) deviation calculated in triplicate.

Ratio of the mean IC_50_ value determined in the normal MCF10a cell line over the mean IC50 value determined in MCF-7 cell line.

Only the selectivity indexes greater than one are referenced.

ND not determined

Peperomin A demonstrated cytotoxic activity against different tested cell lines with selectivity index values greater than 1 for cancer cells (Jokhadze et al., 2007). Peperomin A exhibited cytotoxic activity against MCF-7 with an IC_50_ of 5.58 ± 0.47 µg/mL, compared to a standard drug, Taxol that has an IC_50_ of 3.00 ± 0.07 µg/mL (Table 4). Moreover, peperomin A showed cytotoxic activity against HL-60 and WE-HI cells with IC_50_ values of 16.36 ± 0.62 and 4.62 ± 0.03 µg/mL, respectively. The isolated mixture of phytosterol (stigmasterol and *β*-sitosterol) showed anti-proliferative activity against WEHI-3, HL-60 and MCF-7, with IC_50_ values of 8.06 ± 0.16, 9.84 ± 0.61 and 8.94 ± 0.05 µg/L, respectively, as summarized in Table 3. The *in vitro* cytotoxic activities of different solvent extracts of *P. blanda*, peperomin A and phytosterol reported herein are in accordance with previous reported studies in which, compounds such as secolignans and polyketides, previously isolated from *P. ducluoxii*, showed moderate growth inhibitory activity against VA-13 cells (malignant lung tumor cells) (Li et al., 2007) and secolignans isolated from *P. pellucida*, inhibited the growth of HL-60 (human promyelocytic leukaemia), MCF-7 and HeLa (cervical cancer cells) (Xu et al., 2006). The secolognin and the polyketide isolated from *P. sui* showed cytotoxic activity against HONE-1 and NUGC-3 cell lines (Cheng et al., 2003). Peperomin E showed anti-proliferative activity against human gastric carcinoma SGC-7901, BGC-823 and MKN-45 cell lines, and against non-small-cell lung cancer (NSCLC) cell lines. It also inhibited the proliferation of gastric cells by apoptosis induced via the mitochondrial and PI3K/Akt signalling pathways and exhibited anticancer activity in lung cancer by inhibiting DNMT1 expression and activity (Wang et al., 2016).

In addition, peperomin A and B showed moderate inhibitory effects on HIV-1 IIIB growth in C8166 cells (i.e., a cytopathic effect, CPE) and cytotoxic activity against the C8166 cell line. Previous studies have also reported the chemo protective activity of β-sitosterol in breast and colon cancer cell lines through inhibition of proliferation of the cancer cells, lowering the expression of β-catenin and PCNA, or activating Fas signaling (Awad et al., 2007; Baskar et al., 2010; Chai, Kuppusamy & Kanthimathi, 2008).

Radical scavenging activity using the DPPH assay was tested for all extracts, peperomin A and phytosterol mixture. The inhibitory concentrations (IC_50_) are summarized in Table 5.

**Table 5:** Antioxidant activity by DPPH, FRAP, TPC and TFC.

	DPPH IC_50_ µg/mL	FRAP (M Fe(II)/g)	Total phenolic content (mg GAE/g)	Total flavonoid content (mg QE/g)
Pet. ether	203.80 ± 0.19	162.20 ± 0.80	16.42 ± 1.30	214.30 ± 1.30
DCM	61.78 ± 0.02	381.50 ± 1.31	22.52 ± 0.41	232.00 ± 1.90
MeOH	36.81 ± 0.09	287.00 ± 0.98	31.00 ± 0.44	199.00 ± 1.70
Phytosterol	620.15 ± 10.81	29.94 ± 0.43	ND	ND
BHT	286.64 ± 8.54	1738.80 ± 9.53	ND	ND
VIT C	20.40 ± 0.50	2603.90 ± 10.54	ND	ND
Peperomin A	536.61 ± 6.00	20.79 ± 0.66	ND	ND

The methanol extract exhibited antioxidant activity with an IC_50_ of 36.81 ± 0.09 µg/mL, followed by the dichloromethane extract at 61.78 ± 0.02 µg/mL and 203.80 ± 0.19 µg/mL for the petroleum ether extract. The ferric reducing capability (FRAP) of the three crude extracts was also determined. The extracts exhibited weak ferric reducing ability ranging from 162.2 ± 0.80 to 381.5 ± 1.31 µg/mL, as compared to the standards, vitamin C and BHA, with values of 2603.96 ± 10.54 and 1738.8 ± 9.53 µg/mL, respectively (Table 5).

The peperomin A and phytosterol mixture displayed weak free radical scavenging activity and ferric reducing power indicating that the antioxidant activity recorded in the crude extracts may be due to the presence of other secondary metabolites such as flavonoids and vitamin E that were observed from GCMS and analysis of flavonoid content in the crude extracts. Other reported compounds from *Peperomia*, such as two C-glycosyl-flavones isolated from *P. blanda* (Velozo et al., 2009), phenylpropanoid, benzopyran, chromone, prenylated quinone, secolignan and acylcyclohexane-1, 3-dione could also be responsible for the antioxidant activities observed.

The total phenolic content ranged from 16.42 ± 1.30 mg GAE/g in the methanol extract to 31.00 ± 0.44 mg GAE/g in the petroleum ether extract (Table 5). Moreover, the obtained results showed that total flavonoid content in the dichloromethane extract (232.0 ± 1.9 mg QE/100 g) was higher than in the petroleum ether extract (214.3 ± 1.3 mg QE/g) and the methanol extract (199.0 ± 1.7 mg QE/g). The phenol content varied due to the different solubility of phenol in each solvent extract. The more lipophilic phenols were extracted more readily in petroleum ether and dichloromethane, and their results were similar, while the polar phenols were more readily extracted by methanol. The phenol and flavonoid contents in each solvent extract led to different observations of antioxidant activities. Natural antioxidants like phenolic acids and flavonoids are interesting alternatives to modulate inflammation, and to inhibit the related oxidative processes (Guilhon-Simplicio et al., 2017). Consequently, further isolation of new flavonoids from this plant are warranted for drug discovery.

## Conclusions

The high activities shown by the extracts and the isolated compounds make this plant a valuable source for new anti-cancer drug development and herbal formulations. Further studies should be aimed at exploring and isolating novel bioactive compounds to be used as anti-cancer and anti-oxidizing agents.

##  Supplemental Information

10.7717/peerj.4839/supp-1 Data S1 NMR Spectroscopic data.

10.7717/peerj.4839/supp-2 Supplemental Information 2 Dataset 1 Crystal deposotion files.

## Additional Information and Declarations

### Field Study Permissions

The following information was supplied relating to field study approvals (i.e., approving body and any reference numbers): *Peperomia blanda* (Jacq.) Kunth was collected from the Socotra Island, Yemen (Diksam-sikand), with approval number 120025 and 120026 from the General Directorate of Plant Protection, Yemen, and identified by Dr. Abdul Wali Al-Khulaidi, Department of Botany, Taiz University.

### Data Availability

The following information was supplied regarding data availability: Cambridge Crystallographic Data Centre (CCDC): https://www.ccdc.cam.ac.uk/structures/search?access=referee&searchdepnums=1529062-1529063&searchauthor=Wafa. The raw data are also provided as a Supplemental File.
